# High Performance Capillary Electrophoresis

**DOI:** 10.6028/jres.093.095

**Published:** 1988-06-01

**Authors:** B. L. Karger

**Affiliations:** Barnett Institute, Northeastern University, Boston, MA 02115

Electrophoresis is one of the most powerful tools currently available for the separation of charged species. Its impact in the biological sciences is very great with methods such as SDS polyacrylamide gel electrophoresis, DNA sequencing and two-dimensional gel electrophoresis as standard tools for biological analysis. Typically, electrophoretic methods today involve the use of slab gels or thin layer plates, with detection based on staining and/or blotting procedures. Such approaches are difficult to quantitate and automate and are generally slow. In addition, micropreparative operation involves extraction procedures following separation on the slab gel.

Currently, capillary electrophoresis is being developed as an instrumental approach to electrophoresis. Historically, columns or rods were initially introduced for electrophoretic separations; however, with development of slab gels, the rod technique became less used. However, the above cited limitations of slab gels have rekindled the interest in column operation. High performance capillary electrophoresis bears analogy to HPLC, in terms of injection, column, detection and sample collection. Today, fused silica capillaries of less than 100 µm diameter are typically employed for separation with efficiences in excess of 100,000 theoretical plates.

Equations (1)–(3) present the basic relationships of various parameters with respect to their influence on separation time (*t*), theoretical plates (*N*) and resolution (*Rs*).

$$t=\frac{lL}{\mu_{\text{ep}}V}$$ (1)

$$N=\frac{\mu_{\text{ep}}V}{2D}$$ (2)

$$Rs=\frac{1}{4}\frac{\Delta \mu_{\text{ep}}\sqrt{V}}{\sqrt{2D{\bar{\mu }}_{\text{ep}}}},$$ (3)

where *l* = distance from injection to detector, total capillary length, *V* = applied voltage, µ_ep_ = electrophoretic mobility of the solute, and *D* = solute diffusion coefficient. In these equations, it is assumed that the major cause of band broadening is axial diffusion,

It can be noted that high voltages have a beneficial effect on all three parameters, yielding faster analyses, higher efficiencies and better resolution. The stumbling block toward ever-higher *V* is the Joule heating that accompanies the power generated from these systems (*W* = *I*^2^*R*). We can control to some extent the current generated for a given field by a judicious selection of the buffer in terms of its conductivity and concentration; however, this choice is limited. On the other hand, the tube diameter influences Joule heating, both from the point of view of the number of current carriers and the ability to dissipate the heat generated. Smaller diameter tubes aid in the control of a constant temperature, hence, the use of narrow bore capillaries. A second specific advantage of fused silica is that the wall thickness can be maintained low for improved heat dissipation; indeed, wall thicknesses as small as 30 µm are possible. A polyimide coating on the fused silica permits practical use of narrow wall capillaries. Thus, for a given mobile phase, high fields (*V/L*) and fast separations are possible with fused silica capillaries. Of course, temperature control is important to achieve reproducible separation.

Our laboratory has focused on two approaches in capillary electrophoresis. First, in open capillary operation, we have incorporated chemical selectivity along with high performance separation for resolution of closely related species, Previously, Terabe introduced the use of micelles as a separation medium for the resolution of neutral species in capillary electrophoresis [1]. In this approach, a neutral species partitions within the interior of the micelle, the extent of partition being based on the hydrophobicity of the solute. The micelle moves at a fixed low rate through the capillary, acting as a “moving” stationary phase, in analogy to chromatography. Figure 1 shows a separation of neutral oligonucleotide bases, with solute elution of increasing hydrophobicity [2]. An alternative approach is the use of the surface of the micelle for separation. Figure 2 shows an electropherogram of a polythymidine mixture using SDS and 0.3 mM Cu(II) [2]. The copper is electrostatically attracted to the negatively charged surface of the micelle and differential ligand complexation results in separation. Many chemistries can be considered based on this surface adsorption approach. For example, surfactants with chiral chelates at the polar side can be used for chiral separations. We have separated D,L-dansylated amino acids using such a chelating surfactant and Cu(II). Thus, the selectivity of chromatography can be combined with the efficiency of capillary electrophoresis.

The second approach of our work involved the use of polyacrylamide gel capillary columns to provide a medium analogous to slab gel operation. We have successfully produced stable columns for the separation of important mixtures. Figure 3 shows a separation of myoglobin fragments using the approach of SDS-PAGE [3]. Retention was found to be linear with log *MW*. Indeed, further studies of proteins using a Ferguson plot, revealed a true size separation in these columns [3]. Thus, rapid molecular weight determination and assessment of protein purity is possible using this approach. Figure 3 illustrates another important advantage of this method relative to slab gel operation. Low molecular weight peptides can be separated and quantitated, whereas on slabs they often diffuse off the column.

We have continued work in this area with recombinant materials. Separation of recombinant human growth hormone from a proteolytic clipped variant (2 chain) has been achieved. In addition, the elution of methionine growth hormone (methionine at the N-terminal) has been successful. Other studies have involved oligonucleotides where size separation is again very important. These studies include micropreparative separation of impure mixtures.

Finally, the gel column can be used for entrapment of complexing species to create selective separation, as in affinity electrophoresis. As an illustration of this approach, we have entrapped *β*- cyclodextrin, a neutral cavity-complexation species. This agent has then been used to separate chiral species. The principles of separation have been established and the approaches to achieve optimum separation elucidated.

## Figures and Tables

**Figure 1:**
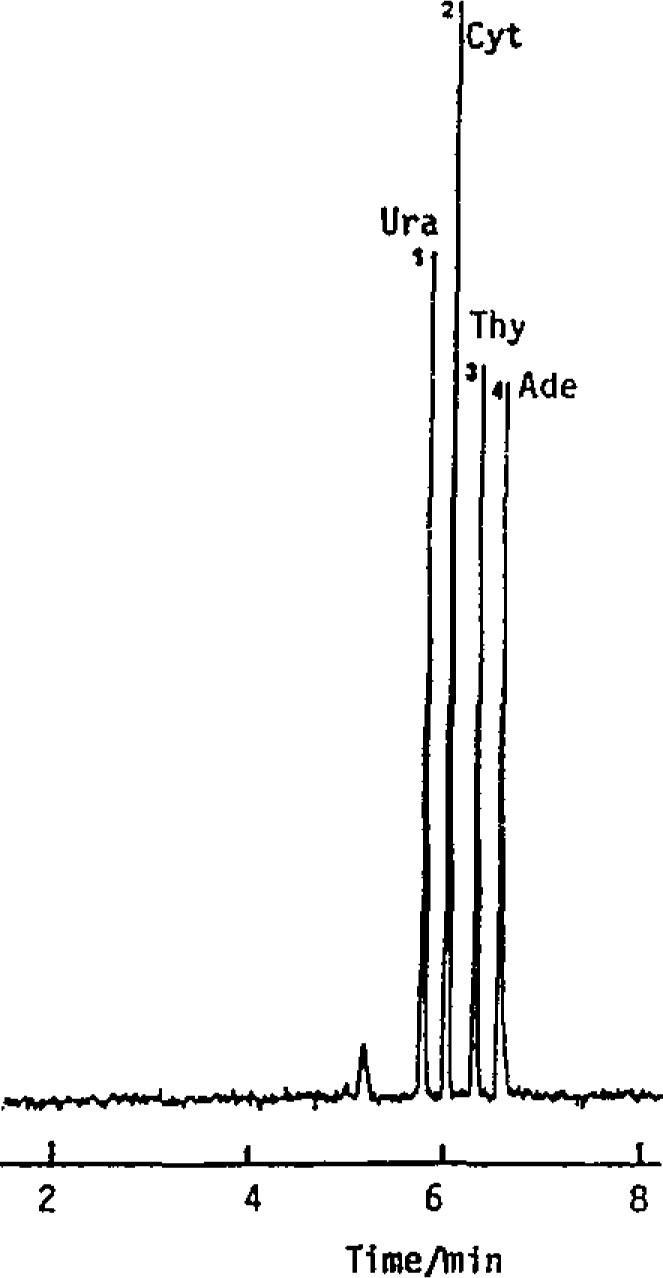
Separation of bases: (1) Ura, (2) Cyt, (3) Thy, (4) Ade. Buffer: 0.025 *M* sodium tetraborate, 0.05 *M* sodium dihydrogen phosphate, 0.1 *M* SDS, pH 7, capillary 650 mm×0.5 mm i.d. Column length 500 mm, applied voltage 14 kV, 50 µA, detection wavelength 210 nm.

**Figure 2:**
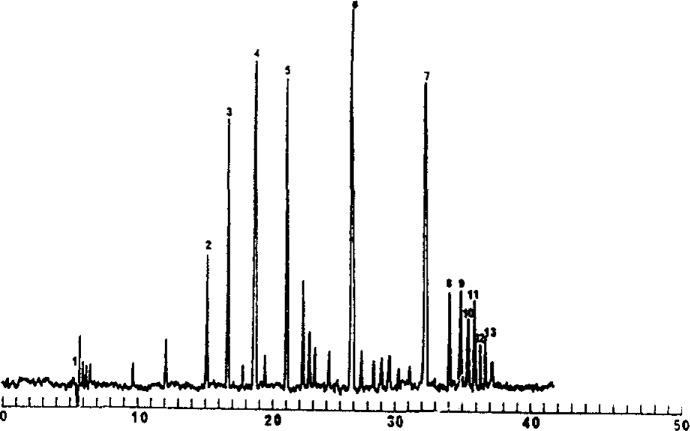
Separation of polythymidine mixture: (1) solvent, (2) d(pT)_2_, (3) d(pT)_3_, (4) d(pT)_4_, (5) d(pT)_6_, (6) d(pT)_10_, (7) d(pT)_12_–d(pT)_18_, (8) d(pT)_12_, (9) d(pT)_14_, (10) d(pT)_15_, (11) d(pT)_16_, (12) d(pT)_17_,(13) d(pT)_18_. 0.3 m*M* Cu(II) added to the buffer. Buffer: 7 *M* urea, 5 m*M* Tris, 5 m*M* Na_2_HPO_4_, pH 7, capillary 650 mm×0.05 mm i.d. Column length 450 mm.

**Figure 3:**
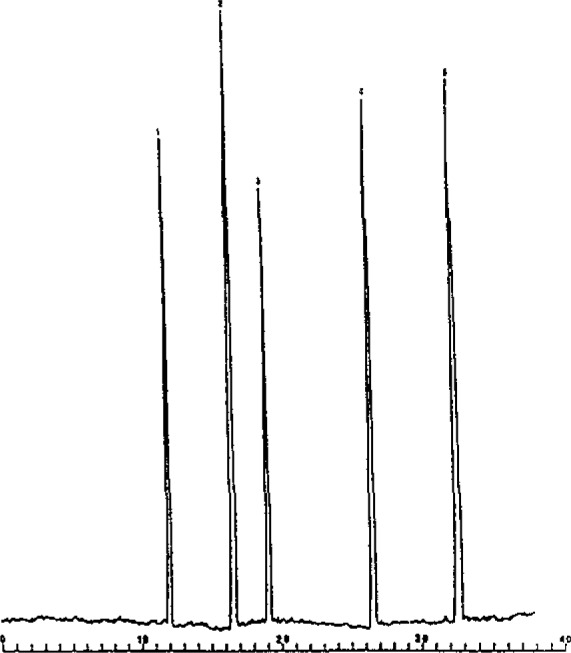
High performance capillary SDS-PAGE separation of myoglobin and several of its fragments. Conditions: 400 V/cm, 34 µA, 25 °C, migration distance 20 cm; fused-silica capillaries; 75 µm ID, T-12.5%, c = 3.3%. Buffer: 0.1 *M* Tris-H_3_PO_4_ (pH = 6.9), 0.1% SDS, 8 *M* urea.
